# Severe acute respiratory syndrome coronavirus-2- or pregnancy-related cardiomyopathy, a differential to be considered in the current pandemic: a case report

**DOI:** 10.1186/s13256-021-02751-3

**Published:** 2021-03-19

**Authors:** Rahim Nejadrahim, Sara Khademolhosseini, Hadiseh Kavandi, Reza Hajizadeh

**Affiliations:** 1 Department of Infectious Diseases, Urmia University of Medical Sciences, Urmia, Iran; 2 School of Medicine, Shiraz University of Medical Sciences, Shiraz, Iran; 3 Connective Tissue Diseases Research Center, Tabriz University of Medical Sciences, Tabriz, Iran; 4 Department of Cardiology, Urmia University of Medical Sciences, Urmia, Iran

**Keywords:** COVID-19, Cardiomyopathy, Pregnancy

## Abstract

**Background:**

There are limited data on cardiovascular complications of coronavirus disease 2019 in pregnancy, and there are only a few case reports on coronavirus disease 2019 related cardiomyopathy in pregnancy. Differentiation between postpartum cardiomyopathy and coronavirus disease 2019 related cardiomyopathy in pregnant women who develop severe acute respiratory syndrome coronavirus-2 infection during peripartum could be challenging. Here, we present a case of possible coronavirus disease 2019 related cardiomyopathy in a pregnant patient, followed by a discussion of potential differential diagnosis.

**Case presentation:**

In this case report, we present the case of a young pregnant Iranian woman who developed heart failure with pulmonary edema after cesarean section. She was treated because of low left ventricular ejection fraction and impression of postpartum cardiomyopathy, and her severe dyspnea improved by intravenous furosemide. On day 3, she exhibited no orthopnea or leg edema, but she was complaining of severe and dry cough. Further evaluation showed severe acute respiratory syndrome coronavirus-2 infection.

**Conclusions:**

The possibility of severe acute respiratory syndrome coronavirus-2 infection should be considered in any pregnant woman who develops cardiomyopathy and pulmonary edema.

## Background

The novel coronavirus disease 2019 (COVID-19), caused by severe acute respiratory syndrome coronavirus-2 (SARS-CoV-2) has become a global health emergency these days. Currently, more than 17.5 million confirmed cases have been reported worldwide [1]. Although COVID-19 is presenting predominantly with pulmonary manifestations, there have been many reports of multiorgan involvement, including the cardiovascular system [2]. COVID-19 related cardiomyopathy, viral myocarditis, myocardial infarction, and arrhythmia are some of the complications reported in the general adult population [3]. Almost 30% of patients with confirmed COVID-19 were found to have evidence of myocardial injury and COVID-19 related cardiomyopathy [4, 5]. The diagnosis of postpartum cardiomyopathy without further evaluation for COVID-19 infection could negatively affect outcomes.

## Case presentation

A 38-year-old pregnant Iranian woman without any history of cardiac disease, diabetes mellitus, hypertension, or psychiatric problems, was admitted for cesarean section because of severe preeclampsia. She did not have any family history of ischemic heart disease or familial cardiomyopathies. She had a bachelor degree in nursery. After successful delivery, she was discharged without any complaints; 12 days after cesarean section, she was admitted again with severe dyspnea and sweating, and her blood oxygen saturation was 80% on room air. Physical examination showed blood pressure of 120/85, heart rate of 115 beats per minute, respiratory rate of 33 breaths per minute, and audible crackles in the lower half of both lungs (day 1 of second admission). Bedside echocardiography showed left ventricular ejection fraction (LVEF) of 40% with a normal left ventricular (LV) size (LV end diastolic size 4.9 cm) with mild to moderate mitral regurgitation. With the diagnosis of pulmonary edema, intravenous furosemide was started, which improved her dyspnea, and with the diagnosis of postpartum cardiomyopathy, bisoprolol, captopril, and furosemide were administered. On day 3, she had no orthopnea or leg edema but was complaining of severe and dry cough. She did not have fever, myalgia, chest pain, or gastrointestinal discomfort. She had no significant medical history and no travel history to a foreign country.

Her vital signs indicated blood pressure of 110/70 mmHg, heart rate of 74 beats per minute, body temperature of 37.2 °C, and respiratory rate of 20 breaths per minute with an oxygen saturation of 94% on room air.

Apart from tachypnea, her physical examination revealed normal jugular venous pulse, scattered bilateral rales, and no peripheral edema. Heart auscultation was normal without pericardial rub.

Diagnostic laboratory tests revealed elevated lactate dehydrogenase (564 U/L) and CRP (3+), leukopenia (WBC count 3400), erythrocyte sedimentation rate (ESR) 50 mm/hour, and mildly elevated serum troponin and D-dimer levels. Other laboratory data are presented in Table 1.

**Table 1:** Laboratory tests of patient on admission

Laboratory test	Value
Blood sugar	172
Urea	30
Creatinin	1.1
Na	139
K	3.5
Hemoglobin	11
Mean corpuscular volume	88.65
Platelets	200,000
Prothrombin time	11.4
Partial thromboplastin time	31
INR	0.92
Cholesterol	134
Triglyceride	272
High-density lipoprotein	35
Low-density lipoprotein	56
SGOT	21
SGPT	27
Creatine phosphokinase	93
Bilirubin (direct)	0.3
Bilirubin (indirect)	0.7

*INR* international normalized ratio, *Na* sodium, *K* potassium, *SGOT* serum glutamic oxaloacetic transaminase, *SGPT* serum glutamic pyruvic transaminase

**Fig. 1:**
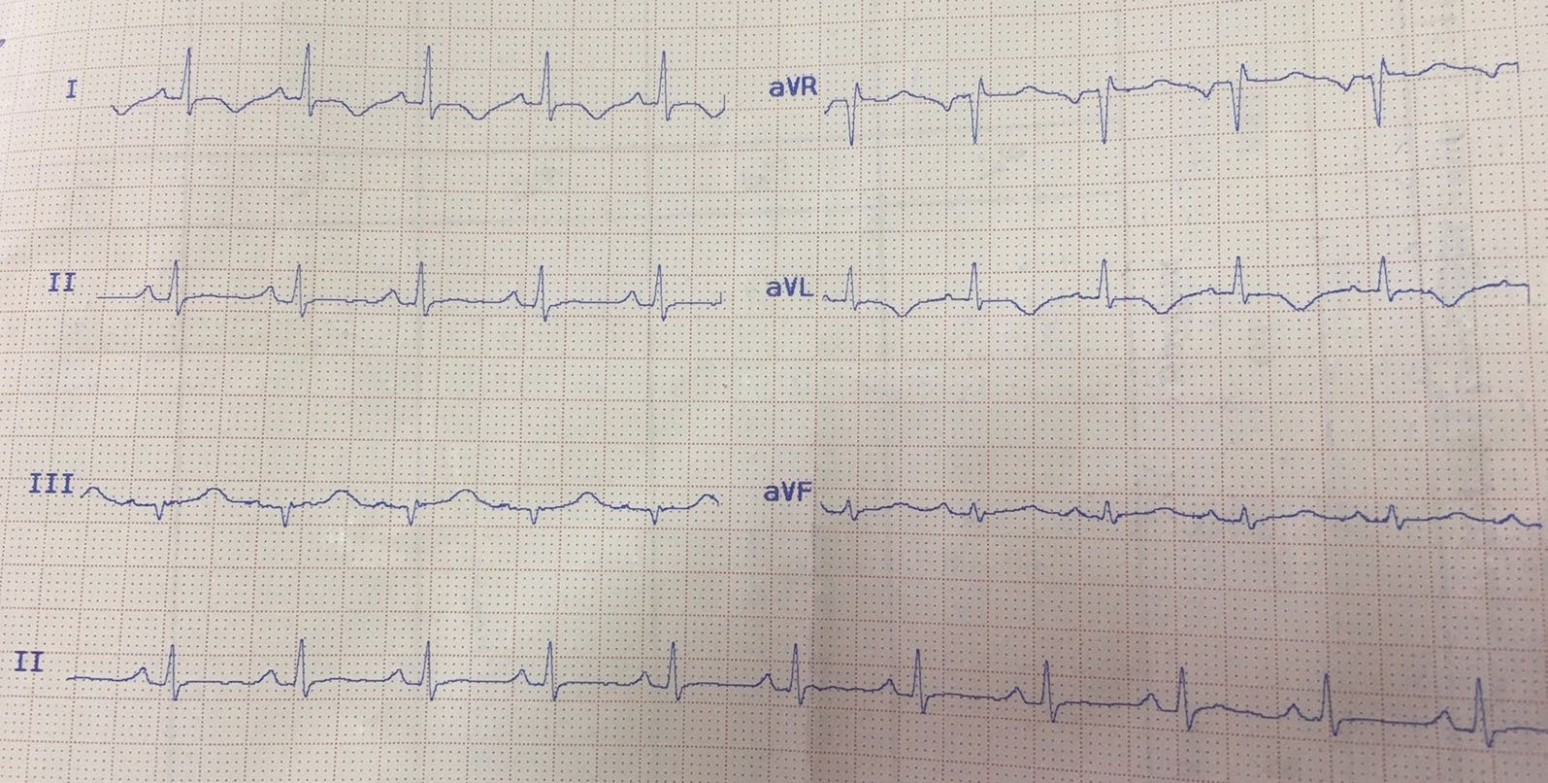
Negative T waves in lateral leads and long QTc

**Fig. 2:**
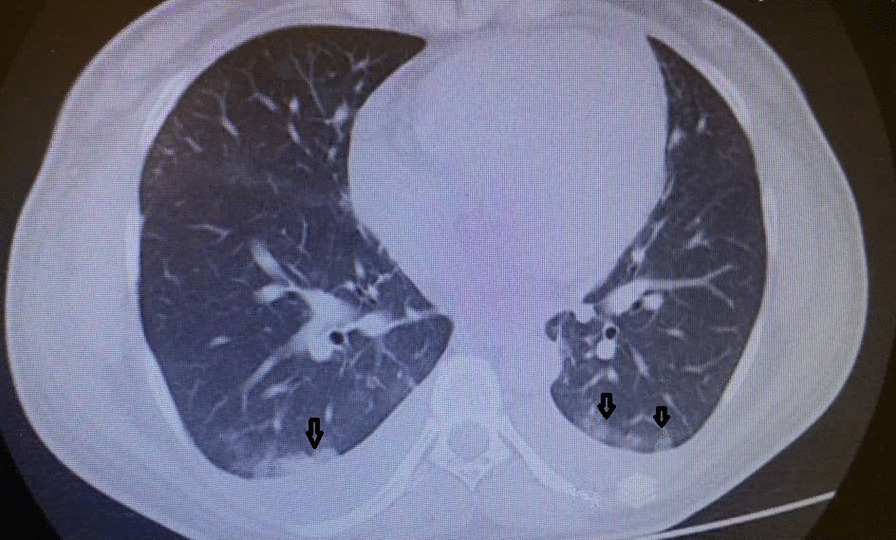
Chest computed tomography showing peripheral patchy and ground-glass opacities (arrows) in both lungs and pleural effusion

A 12-lead electrocardiogram showed negative T waves in I, AVL, and V5–V6 leads and poor R progression in precordial leads without Q wave and with prolonged QT interval (QTc 500 ms) (Fig. 1). Because her tachypnea did not resolve with proper furosemide administration, on day 3, a new chest X-ray was done that was not consistent with pulmonary edema. To evaluate other causes of dyspnea, a chest computed tomography was done, showing peripheral patchy and ground-glass opacities in both lungs concordant with COVID 19 infection. No hilar or mediastinal lymphadenopathy was observed. There was also pleural effusion on both sides (Fig. 2).

A nasopharyngeal swab for severe acute respiratory syndrome coronavirus 2 (SARS-CoV-2) real-time reverse transcription polymerase chain reaction (RT-PCR) was positive. Thus, azithromycin, lopinavir–ritonavir, subcutaneous interferon β1 (Resigen), and intravenous immunoglobulin gamma (IVIG) (20 g/day for 3 days) were added to her medications [6].

**Fig. 3.**
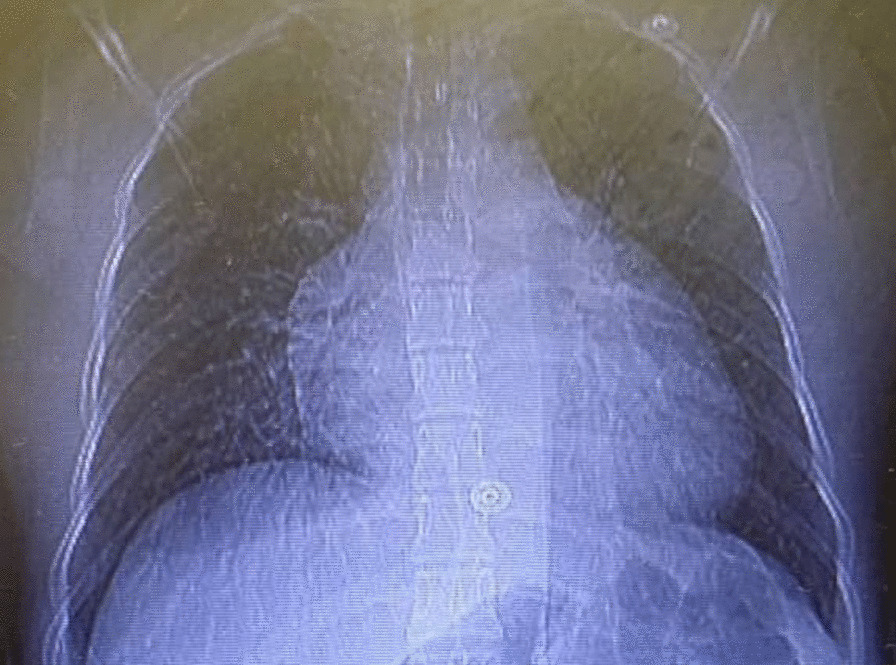
Chest X-ray showing improved COVID-19 infection and no pulmonary edema with cardiomegaly on 12th day of admission

On the eighth day of second admission, she again developed severe dyspnea and pulmonary edema, and her blood pressure was 110/70, heart rate 110 beats per minute, respiratory rate 25 breaths per minute, and audible crackles in the lower third of both lungs. Intravenous furosemide 40 mg stat and 6 mg per hour started, which stabilized her condition. Echocardiography was performed again on the 11th day of admission, showing LVEF of 30%, global hypokinesis, LV enlargement (LV end diastolic size 5.8 cm), and normal right ventricle (RV) size with reduced RV systolic function. Again, IVIG 20 g/day was started and continued for 3 days. On the 12th day of admission, the patient’s clinical status stabilized and her symptoms disappeared. Chest X-ray showed improvement of lung infection and no evidence of pulmonary edema (Fig. 3). Her heart rate decreased to 70 beats per minute with a respiratory rate of 14 breaths per minute. Oxygen saturation on room air was 95%, and her body temperature was 37.0 °C (by mouth).

Her second RT-PCR was negative, and she was discharged with a prescription of carvedilol, enalapril, furosemide, digoxin, and bromocriptine.

Thirty days after discharge, she was admitted to the clinic as an outpatient. At this visit, her vital signs were stable, she had mild exertional dyspnea, and her O_2_ saturation was 96% on room air. She was excited about starting her job as a nurse, following with necessary COVID 19 prevention guidelines.

## Discussion

Cardiovascular complications of COVID-19 are important in the prognosis and survival of patients. Guo *et al*. studied 187 COVID-19 patients and found 27.8% with evidence of myocardial injury [4]. A study of the Washington State COVID-19 cohort revealed cardiomyopathy in 33% of nonpregnant patients in the intensive care unit [5]. There are limited data on COVID-19 in pregnancy. Juusela and colleagues reported two cases of COVID-19 related cardiomyopathy in pregnant women [7].

The mechanism by which SARS-CoV-2 causes myocardial injury is not fully understood. Studies on the role of ACE-2 receptors in viral life cycle and the effects of ACE-2 disruption show that the interaction between ACE-2 and SARS virus might be causing myocardial inflammation and injury [8]. Additionally, the downregulation of ACE-2 hinders the cardioprotective effects of angiotensin and subsequently increases tumor necrosis factor alpha (TNFα) production, which may be contributing to myocardial damage [9]. Exaggerated cytokine response is also a potential mediator of cardiomyocyte damage [4].

Here, our case was a young female with no remarkable risk factors for cardiomyopathy. The main cause of her disease could be undiagnosed postpartum cardiomyopathy, aggravated by COVID-19 cardiomyopathy. According to Pierce-Williams *et al*. [10], who studied severe and critical COVID-19 in a cohort of hospitalized pregnant patients in the United States, only 17% of patients had preexisting cardiac disease. Being of childbearing age, in addition to a lack of other cardiac risk factors, makes the undiagnosed heart failure a less likely differential diagnosis.

Pregnancy-associated cardiomyopathy is a major differential diagnosis. It is defined as the development of heart failure toward the end of pregnancy or closely after delivery with the absence of another identifiable etiology for heart failure [11]. The exact mechanism is still unclear and probably multifactorial. Angiogenic imbalance, as evidenced by prolactin levels, and high levels of inflammatory cytokines such as TNFα and interleukin-6 (IL-6) are potential contributors [12]. High levels of inflammatory markers, especially IL-6, might be a clue to finding a possible common pathophysiology for pregnancy-associated cardiomyopathy and COVID-19 related cardiomyopathy. IL-6 also plays an important role in COVID-19 pathophysiology. Zeng *et al*. reviewed inflammatory markers in over 3900 COVID-19 patients and found that survivors had a lower level of IL-6 compared with nonsurvivors [13]. It is possible that common pathways such as IL-6 could trigger and aggravate dilated cardiomyopathy in pregnant women who develop COVID-19 in peripartum period, and COVID-19 prevention might be more important in this period. Although rapid progression of heart failure in postpartum dilated cardiomyopathy is not uncommon,it is important to observe the severity of dilated cardiomyopathy in peripartum COVID-19-positive patients to better understand its nature [14, 15].

Further studies are needed to investigate whether pregnancy affects the chance of developing COVID-19-related cardiomyopathy compared with the general population. Also, there is a lot to learn about how pregnancy and COVID-19-related cardiomyopathy affect each other. Studying the role of inflammatory cytokines such as IL-6 in the process of myocardial injury might shed light on the pathophysiology underlying pregnancy and COVID-19-related cardiomyopathy. A dedicated subgroup analysis of myocardial injury in ongoing trials on anti-IL-6 receptor antibodies, such as tocilizomab, for COVID-19 is recommended.

We suggest routine echocardiographic evaluation of pregnant patients with COVID-19 as they are at a higher risk of cardiovascular complications due to the role of inflammatory markers.

## Conclusion

It is important to test for severe acute respiratory syndrome coronavirus-2 infection in pregnant women with cardiomyopathy and primary diagnosis of postpartum cardiomyopathy during the COVID-19 pandemic.

## Data Availability

All data and material collected during this study are available from the corresponding author upon reasonable request.

## Abbreviations

SARS: Severe acute respiratory syndrome
LVEF: Left ventricular ejection fraction
CRP: C-reactive protein
WBC: White blood cells
IVIG: Intravenous immunoglobulin
RV: Right ventricle
ICU: Intensive care unit
ACE: Angiotensin-converting enzyme
IL-6: Interleukin 6
